# Reliability of two goniometric methods of measuring active inversion and eversion range of motion at the ankle

**DOI:** 10.1186/1471-2474-7-60

**Published:** 2006-07-28

**Authors:** Collette Menadue, Jacqueline Raymond, Sharon L Kilbreath, Kathryn M Refshauge, Roger Adams

**Affiliations:** 1 School of Physiotherapy, University of Sydney, Sydney, Australia

## Abstract

**Background:**

Active inversion and eversion ankle range of motion (ROM) is widely used to evaluate treatment effect, however the error associated with the available measurement protocols is unknown. This study aimed to establish the reliability of goniometry as used in clinical practice.

**Methods:**

30 subjects (60 ankles) with a wide variety of ankle conditions participated in this study. Three observers, with different skill levels, measured active inversion and eversion ankle ROM three times on each of two days. Measurements were performed with subjects positioned (a) sitting and (b) prone. Intra-class correlation coefficients (ICC_[2,1]_) were calculated to determine intra- and inter-observer reliability.

**Results:**

Within session intra-observer reliability ranged from ICC_[2,1] _0.82 to 0.96 and between session intra-observer reliability ranged from ICC_[2,1] _0.42 to 0.80. Reliability was similar for the sitting and the prone positions, however, between sessions, inversion measurements were more reliable than eversion measurements. Within session inter-observer measurements in sitting were more reliable than in prone and inversion measurements were more reliable than eversion measurements.

**Conclusion:**

Our findings show that ankle inversion and eversion ROM can be measured with high to very high reliability by the same observer within sessions and with low to moderate reliability by different observers within a session. The reliability of measures made by the same observer between sessions varies depending on the direction, being low to moderate for eversion measurements and moderate to high for inversion measurements in both positions.

## Background

Physiotherapists measure active inversion and eversion ankle range of motion to evaluate the severity of ankle dysfunction and to monitor treatment outcomes. Although visual estimation is commonly used in the clinic, it is unlikely to be sufficiently reliable to enable clinicians to confidently either monitor progress between treatment sessions, or compare measurements between clinicians. For example inter-observer reliability for visual estimation of active plantarflexion and dorsiflexion, the only movements for which data are available, is low (plantarflexion, ICC = 0.48; dorsiflexion, ICC = 0.34) [1]. It is therefore recommended that objective measurements be made, and goniometers are the simplest tool available. Two goniometric measurement protocols have been described for the measurement of inversion and eversion range: one in prone and one in either supine or sitting. In prone, measurements are taken from the posterior aspect of the foot whereas in sitting or supine, measurements are taken from the anterior aspect of the foot [2]. While both methods are commonly used for measuring ankle inversion and eversion range in the clinic, the reliability of these measurements is unknown.

To be useful in the clinic and for research, a protocol for goniometric measurement of inversion and eversion needs to be highly reliable, both within and between observers, and should provide accurate information about ankle motion. Of particular relevance is reproducibility of the technique across measurement occasions, and whether measurements are reproducible among clinicians, regardless of their experience. Reliability of passive inversion and eversion movements has been investigated in two studies [3,4], however, active movements are most commonly assessed in the clinic to monitor impairments. The reliability of goniometric measurements of active inversion and eversion ankle range has not been fully evaluated. Only one study has examined the reliability of goniometric measurements, evaluating active inversion but not eversion, in sitting, finding that inter- and intra-observer reliability was moderate with a correlation of 0.69 and 0.795, respectively [5]. However, the authors did not report the reliability coefficient used, yet this is important because Pearson's r can overestimate reliability [6,7], and some forms of intraclass correlation coefficients cannot be generalised to other observers [8]. Furthermore, observers were not blinded to the measurements and consequently expectation bias cannot be excluded.

The present study aimed to investigate the reliability of two protocols for goniometric measurements of active ankle inversion and eversion range of motion (ROM), and the reliability of three examiners with different levels of training and experience. Specifically, this study measured within and between session intra-observer reliability and within session inter-observer reliability of two goniometric protocols. Additional questions of interest were whether reliability was better with one particular protocol and whether the training and experience of the observer affected reliability. A secondary aim of this study was to compare goniometry to a reference standard for measuring total ankle inversion-eversion ROM.

## Methods

### Subjects

Thirty-one subjects, 20 females and 11 males aged between 21 and 59 years (mean 35.4 years) volunteered to participate in the study. Subjects were excluded if they had sustained an ankle injury within 4 weeks prior to testing, or between test sessions (N = 1 ankle). Active inversion and eversion range of motion was therefore measured in both ankles of 30 subjects (N = 60 ankles). Eleven of the original 31 subjects (16/62 ankles; 35%) had a past history of at least one ankle injury. Injuries included inversion sprain (N = 7 ankles), plantar fasciitis (N = 1), malleolar fracture (N = 1), peroneal tendonitis (N = 1), and a traumatic accident (N = 1) resulting in bilateral compartment syndrome and unilateral metatarsal fractures. The study was approved by The University of Sydney Human Ethics Committee and consent was obtained from all subjects prior to commencement of data collection.

### Reliability of inversion and eversion measurements

#### Observers

Three observers measured active inversion and eversion range of movement with a Universal goniometer in all subjects. Observer 1 was an exercise scientist with three years experience in ankle goniometry but no formal musculoskeletal training, Observer 2 was a manipulative physiotherapist with 25 years of clinical experience, and Observer 3 was a fourth year physiotherapy student considered to be a relative novice at ankle goniometry.

#### Measurement procedure

Measurements were made on two occasions between one and two weeks apart. On each test occasion, active inversion and eversion range was measured 3 times by each observer in both the sitting and prone positions. Subjects completed four repetitions of full range active inversion and eversion cycles prior to the commencement of measurements as pre-conditioning to minimise the effects of creep [9].

**Figure 1:**
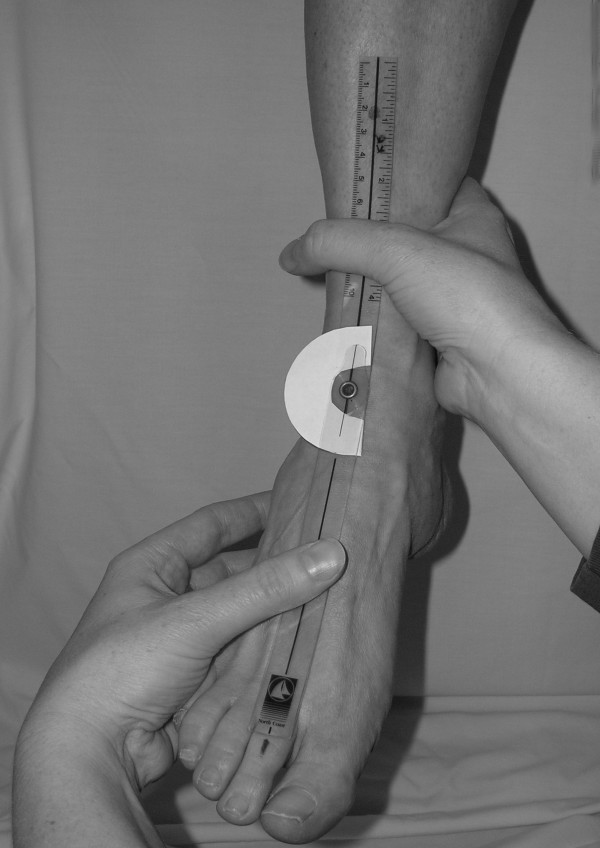
**Goniometric measurement in the sitting position**. Anterior view of the alignment of the small shielded goniometer with the subject positioned in sitting. The ankle was relaxed in comfortable plantarflexion.

**Figure 2:**
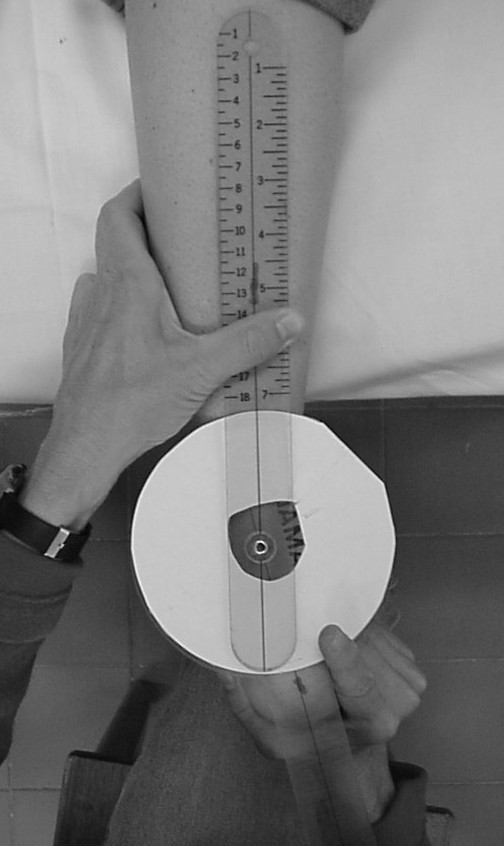
**Goniometric measurement in the prone position**. Aerial view of the alignment of the large shielded goniometer with the subject positioned in prone. The observer's right hand is in position to maintain the plantargrade position during inversion and eversion movements.

Order of testing the following variables was randomised for each subject: goniometry and Fastrak measurements; the sitting and prone positions; inversion and eversion direction of movement in each position; and the order of observers for goniometric measurements. To minimise bias, observers were blinded to all measurements on each test occasion by placing a shield over the measurement scale on the goniometer (Figures 1 and 2). A fourth researcher read and recorded all goniometric measurements. Accuracy of the measurements was enhanced by placing the goniometer on an enlarged circle (diameter 25.5 cm) with degrees clearly marked. Scores were recorded as whole degrees, scores of 0.5° and above being recorded as the next highest whole degree. On the second test occasion the recorder was blinded to recordings from the first test occasion to prevent potential bias. Observers were not informed of their performance until data collection was complete.

#### The two test positions

Active inversion and eversion movements were measured in two positions: sitting and prone lying. Each observer positioned subjects and located and marked the bony landmarks for goniometric alignment using the methods described by Norkin and White [2]. Landmarks were palpated on each ankle and marked with a non-permanent marker by each observer. These marks were removed before the next observer began taking measurements.

##### (a) Sitting position

Subjects were seated on the edge of a plinth with the lower leg over the bed unsupported, and the ankle in a comfortable relaxed position, usually in some plantarflexion (Figure 1). Landmarks used were: the midpoint between the malleoli on the anterior aspect of the ankle; the midline on the anterior aspect of the lower leg using the crest of the tibia as a reference point; and the longitudinal midline on the anterior surface of the second metatarsal [2]. Subjects moved from a self-selected neutral position actively to the end of range guided by the observer, however the amount of plantarflexion during measurements was not controlled. We used a small Universal goniometer with arm length 17 cm from axis to tip.

##### (b) Prone position

Subjects were positioned in prone with the lateral malleolus extended approximately 10 cm over the end of the plinth (Figure 2). The ankle joint was held in plantargrade. Landmarks used were: the midpoint between the malleoli on the posterior aspect of the ankle; the midline on the posterior aspect of the lower leg; and the midline of the posterior aspect of the calcaneus [2]. During measurement the talocrural joint was maintained in plantargrade by manual guidance from the observers. We used a large Universal goniometer with arm length 31.5 cm from axis to tip to measure the movement.

### Comparison to a reference standard

Because there is no non-invasive gold standard available to measure ankle motion, we used a reference standard, the 3SPACE Fastrak electromagnetic tracking system (Polhemus, Colchester, Vermont), to gain measures of the magnitude of range of ankle inversion and eversion. The Fastrak system is an electromagnetic tracking device that describes the three-dimensional position and orientation of a sensor relative to a source [10]. This technique is non-invasive and has high accuracy, test-retest reliability and face validity [10].

On the first test occasion, active total inversion-eversion range was measured using the Fastrak. Measurements were made in the same sitting and prone positions used during goniometric measurements and the same observer made all Fastrak measurements for all subjects.

Twenty seconds of data were collected from 2 electromagnetic sensors during three continuous cycles of active inversion-eversion in all test ankles. The sensors were attached to the test ankle using tape. One sensor was attached to the lateral malleolus (the source sensor) and the second sensor was attached to the lateral aspect of the calcaneus. The sensors were aligned so that the leads were parallel to one another. Signals were sampled at 60 Hz during all recording procedures. The system was linked to a personal computer that controlled the acquisition and storage of data. Software developed at The University of Sydney interpreted the raw kinematic data generated by the Fastrak system. The position and orientation of the tracking sensors were described in degrees relative to the cardinal planes of the body.

### Data analysis

Descriptive statistics (mean ± SD) were calculated for goniometric measurements of active inversion, eversion and total inversion-eversion movements, and for Fastrak measurements of total range in both the sitting and prone positions. Intra-observer and inter-observer reliability of goniometric measurements of active ankle inversion and eversion were determined using intraclass correlation coefficients (ICC_[2,1]_) with 95% confidence intervals [8]. To determine intra-observer reliability, ICC_[2,1] _was calculated for each observer using the three measurements of each movement direction, in each position during session 1 for within session reliability, and for the first of the 3 measurements on each day for between session reliability. Within session inter-observer reliability was determined by using the first of the three measurements in each direction and for each protocol made by each tester from session 1. The strength of correlation was interpreted using the classification scheme of Munro [11], ie. 0–0.25 being "little if any", 0.26–0.49 being "low", 0.50–0.69 being "moderate", 0.70–0.89 being "high" and 0.90–1.00 being "very high" correlation. The standard error of the measurement (SEM) and 95% confidence intervals were calculated to provide an estimate of the amount of error associated with the measurement in the same units as the measurement [12,13].

A 3-way analysis of variance was used to determine whether there was a significant difference in reliability among position, direction and measurement occasions for inter-observer and intra-observer ICC score. The within-subject factors were position (sitting or prone), direction (inversion or eversion) and measurement occasion (session 1 or 2).

The relationship between sitting and prone goniometric measurements was determined by calculating the Pearson's product moment correlation (r) for all comparisons. The coefficient of determination (r^2^) was used to determine the proportion of total variance in prone measurements that could be explained by sitting measurements. The coefficient of determination (r^2^) was also calculated to express the relationship between goniometric and Fastrak measurements, using the average total inversion-eversion range recorded by each observer on the first measurement occasion, and for all observers, and the average total range recorded by the Fastrak system. All analyses were conducted using the statistical software package, SPSS Version 10.0. Significance level was set at p < 0.05.

## Results

The average range of inversion and eversion motion measured on day 1 was similar to average range measured on day 2 by each observer, and among observers (Table 1), although the magnitude of the range of motion differed between positions. Total inversion-eversion ROM was 43.1 ± 10.1° for sitting and 24.2 ± 6.4° for the prone position. The total inversion-eversion ROM obtained with Fastrak was 23.1 ± 6.9° in sitting and 24.2 ± 7.3° in prone.

### Within-session reliability

Within session intra-observer reliability was high to very high, ranging from ICC_[2,1] _= 0.82 to 0.96 (Table 2). There was no significant difference in ICC_[2,1] _scores between positions (p = 0.94) or between directions of movement (p = 0.22). Inter-observer reliability was significantly higher (p = 0.002) in sitting than in the prone position: in sitting, reliability was moderate to high and in the prone position reliability was low to moderate (Table 2). Inversion measurements were significantly more reliable than eversion measurements (p = 0.004).

The intra-observer and inter-observer standard error of measurement (SEM) and 95% confidence level (SEM × 2) for goniometric measurements of inversion and eversion is presented in Tables 3 and 4. Within a test session, the error associated with a single observer making either an inversion or eversion measurement was between 4° and 6° in the sitting position and between 2° and 3° in the prone position (Table 3). Within a test session, the inter-observer measurement error associated with making either an inversion or eversion measurement was 9° in the sitting position and 6° to 8° in the prone position (Table 4).

### Between-session reliability

Between session intra-observer reliability ranged from low to high (ICC_[2,1] _= 0.42 to 0.80: Table 5). Between sessions, there was no difference in the intra-observer reliability using the sitting protocol compared with the prone protocol (p = 0.69), however, raters were more reliable when measuring inversion than eversion (p = 0.008). Between test sessions (Table 6), the measurement error in the sitting position ranged from 7° to 11° and from 4° to 8° in the prone position.

### Relationship between sitting and prone measures

Goniometric measurements of inversion were moderately well correlated between the prone and sitting positions (Pearson's r = 0.49 to 0.60 for three observers). For the average goniometric measurement, 42% of sitting inversion predicted the prone inversion score. For eversion, there was no significant correlation between prone and sitting measurements, with Pearson's r consistently near zero for all observers.

### Comparison of goniometry to a reference standard

Goniometric measurements of active inversion-eversion total range in sitting were moderately well correlated with Fastrak measurements in sitting, ranging from r = 0.52 to 0.58 for individual observers. In prone, however, low correlations were demonstrated between measurements, ranging from r = 0.36 to 0.48 for individual observers. For Fastrak measurements made in sitting, 32% of the variance could be explained by the goniometric measurements whereas 18% of the variance in Fastrak measurements made in prone could be explained by the goniometric measurements.

## Discussion

We found that the reliability of goniometry for the measurement of active ankle inversion and eversion range of motion was very variable for both the sitting and prone measurements. However, our findings also suggest that, for an individual clinician using a standardized method, the measurement of inversion and eversion is highly reproducible within a session, although reliability was variable between sessions. Inversion measurements were more reliable than eversion measurements. When compared to a reference standard, measurements of both inversion and eversion using a goniometer were moderately related in the sitting position, but poorly related in the prone position. However, there was an average discrepancy of almost 20° between absolute angles for the reference standard and measurements made in the sitting position, suggesting that the sitting position does not provide an accurate absolute measure of ankle range of motion.

Measurements were made on both ankles, despite potential interdependence of the leg measurements. We believed the impact of interdependence to be small because more than one third of participants had a unilateral injury or bilateral injuries of various types, and therefore range of motion was likely to be independent in these participants. It is also likely that measurement of right and left feet is independent, eg, depending on hand dominance of the observer. We thus retained the measurements as independent measures, although pairs of measurements were taken from each subject.

The sitting position produced consistently larger scores for inversion and eversion range of motion than the prone position (Table 1). This can be explained because in sitting, the measurement is made from the anterior aspect of the ankle and foot, and thus involves both ankle and forefoot motion whereas in prone the measurement is made from the posterior aspect of the ankle and foot, and involves ankle motion only. In this study, although we were interested in inversion and eversion at the ankle joint, in the sitting position, the location of the landmarks and the fact that range of motion was measured from an anterior aspect, meant that the inversion measurement obtained involved supination ie. a combination of plantarflexion, inversion and adduction. The eversion measurement involved a combination of pronation, dorsiflexion, eversion and abduction. Therefore, the measurement obtained was a combination of movements at the ankle (talocrural and subtalar joints) and the tarsal joints. In contrast, measurements performed in the prone position were restricted to talocrural and subtalar joint motion only.

One of the most significant features of this study is that it presents for the first time a comprehensive assessment of the reliability of a commonly used clinical measure. Our results are consistent with those of the only other study [5] to report intra-observer reliability, finding reliability of inversion measurements across four sessions to be 0.795. This score compares favorably with our score for intra-observer reliability across two measurement sessions of 0.83.

In clinical practice, goniometry is used to assess changes in range of motion due to treatment. Therefore, of primary interest to clinicians is how reproducible measurements are when taken across two sessions. Secondly, there is clinical interest in sensitivity of detecting real changes in ankle ROM. Based on our observations, if taken by the same examiner, the clinician can expect low to high reliability across two measurement sessions. The position in which the measurements are made makes no difference to reliability, nor does the experience or the training of the observer, as long as they are familiar with the technique. However, reliability is generally lower for eversion movements.

To detect a true change in ankle ROM under the best conditions ie. the same observer repeating measures of inversion ROM, a change of at least 7° is necessary in the sitting position and 6° in the prone position to be confident that a true change in ROM has occurred. These changes are larger than previously suggested conservative estimates of a clinically relevant change in joint range [7]. Thus, goniometry is useful for measuring inversion and eversion range of motion at the ankle, but measurements are more reliable for inversion using the test protocols, and are more reliable in the sitting position. Although measurements of eversion were less reliable than measurements of inversion, reliability was moderate, and thus should still be considered a useful tool.

### Sources of measurement error

**Figure 3:**
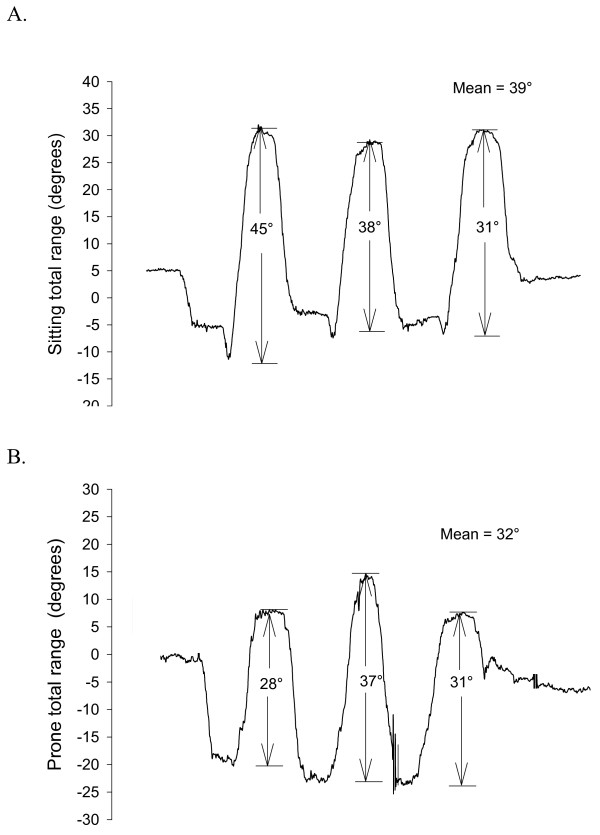
**Fastrak measurements in (a) sitting and (b) prone of three cycles of active inversion-eversion range**. Traces show three cycles of active inversion-eversion total range of motion. Data are from a single, representative subject.

Several sources of error may have contributed to the scores that we observed. First, the identification of landmarks between measurement occasions may have been inconsistent, a problem that may have been greater in the prone position where there were no bony reference points to assist landmark identification [7]. Inconsistencies in performance of the movement on the subject's part may have also contributed to measurement error. Data from the Fastrak measurements demonstrate that subjects were not entirely consistent when performing 3 repeat movements (Figure 3). That is, while the subject was instructed to move through full range, it appears that this was not consistently executed over the 3 repeat measurements. Therefore, some of the variation recorded both within an observer's measurements and between the measurements of different observers could be attributed to the inconsistent movement by subjects. For example, within a session one observer may have measured 6°, 4°, then 7° of inversion. While this would suggest that the measurements taken by that observer were not consistent, it may also be possible that the subject was not performing the movement consistently.

### Comparison with reference standard

The complexity of the ankle joint is likely to reduce accuracy because a multi-planar joint movement was measured using a goniometer, a device that records two-dimensional movement, whereas the Fastrak system recorded movement in all three planes. This would explain, in part, the discrepancy between measurements made with a goniometer and the reference standard. Goniometric measurements of joint range require that the goniometer axis be aligned with the joint axis [2]. However, the subtalar joint axis is oblique, crosses three planes, and changes orientation during inversion and eversion movements [14,15]. Therefore, it is likely that the observer cannot accurately align the goniometer axis with the subtalar joint axis throughout the entire range, and thus reduced the accuracy of goniometric measurements.

## Conclusion

Overall, reliability of standardized measurement of ankle inversion and eversion range of motion was very variable within observers, between observers and between sessions. Intra-observer reliability was high to very high within a test session, and between-session reliability was highest for inversion range of motion.

## Competing interests

The author(s) declare that they have no competing interests.

## Authors' contributions

CM participated in the design of the study, data collection, analysis and drafting the manuscript. JR conceived the study, participated in the design of the study, data collection and writing the manuscript. SK conceived the study, participated in the design of the study, data collection, analysis and commenting on various drafts of the manuscript. KR conceived the study, participated in the design of the study, data collection, analysis and commenting on various drafts of the manuscript. RA participated in the design of the study and data analysis. All authors read and approved the final manuscript.

## Pre-publication history

The pre-publication history for this paper can be accessed here:



## Figures and Tables

**Table 1:** Range of inversion and eversion motion (mean ± SD) for each observer on each day. The range for each observer is an average of the 3 trials for all 60 ankles.

	Sitting		Prone	
	Inversion	Eversion	Inversion	Eversion
Day 1				
Observer A	32.9 ± 8.3	11.0 ± 7.2	13.6 ± 6.0	7.7 ± 4.0
Observer B	29.5 ± 8.8	12.0 ± 6.9	17.6 ± 6.5	9.0 ± 3.6
Observer C	32.1 ± 9.2	10.4 ± 8.0	13.8 ± 4.9	8.1 ± 3.2
Mean ± SD for all observers	31.5 ± 8.8	11.1 ± 7.4	15.0 ± 6.1	8.3 ± 3.6
				
Day 2				
Observer A	34.6 ± 7.5	8.2 ± 6.2	15.1 ± 5.1	9.0 ± 3.0
Observer B	31.2 ± 8.7	12.1 ± 7.3	17.5 ± 7.0	9.2 ± 3.8
Observer C	33.0 ± 8.7	9.5 ± 8.8	14.9 ± 4.9	8.8 ± 3.0
Mean ± SD for all observers	32.9 ± 8.4	9.9 ± 7.6	15.8 ± 5.8	9.0 ± 3.3

**Table 2:** Within-session reliability. Data are presented for ICC _2,1 _(95% CI)

		Sitting		Prone	
	Observer	Inversion	Eversion	Inversion	Eversion

Intra-observer	A	0.92 (0.88 to 0.95)	0.90 (0.86 to 0.94)	0.94 (0.91 to 0.96)	0.94 (0.91 to 0.96)
	B	0.91 (0.86 to 0.94)	0.82 (0.75 to 0.88)	0.94 (0.91 to 0.96)	0.83 (0.76 to 0.89)
	C	0.96 (0.93 to 0.97)	0.93 (0.90 to 0.96)	0.94 (0.91 to 0.96)	0.88 (0.82 to 0.92)
					
Inter-observer		0.73 (0.61 to 0.82)	0.62 (0.49 to 0.74)	0.54 (0.33 to 0.70)	0.41 (0.25 to 0.56)

**Table 3:** Within session, intra-observer standard error of measurement (SEM) and 95% confidence level (SEM × 2)

		Sitting		Prone	
Observer		Inversion	Eversion	Inversion	Eversion

A	SEM	2.1	2.1	1.4	1.0
	SEM × 2	4	4	3	2
B	SEM	2.6	2.9	1.6	1.4
	SEM × 2	5	6	3	3
C	SEM	1.9	2.2	1.2	1.1
	SEM × 2	4	4	2	2

* SEM = sd√ (1-ICC[2,1]).

** SEM × 2 indicates the extent of measurement error associated with a single measurement i.e. the range within which the true score lies with a 95% confidence level.

**Table 4:** Within-session, inter-observer standard error of measurement (SEM) and 95% confidence level (SEM × 2).

	Sitting		Prone	
	Inversion	Eversion	Inversion	Eversion

SEM	4.6	4.5	4.1	2.8
SEM × 2	9	9	8	6

* SEM = sd√ (1-ICC[2,1]).

** SEM × 2 indicates the extent of measurement error associated with a single measurement i.e. the range within which the true score lies with a 95% confidence level.

**Table 5:** Between session, intra-observer reliability

	Sitting		Prone	
	Inversion	Eversion	Inversion	Eversion

A	0.80 (0.66 to 0.88)	0.42 (0.18 to 0.61)	0.53 (0.32 to 0.69)	0.60 (0.37 to 0.75)
B	0.62 (0.43 to 0.75)	0.44 (0.20 to 0.62)	0.76 (0.63 to 0.85)	0.60 (0.42 to 0.75)
C	0.78 (0.66 to 0.86)	0.64 (0.47 to 0.77)	0.64 (0.46 to 0.77)	0.54 (0.34 to 0.70)

**Table 6:** Between-session, intra-observer standard error of measurement (SEM) and 95% confidence level (SEM × 2).

		Sitting		Prone	
Observer		Inversion	Eversion	Inversion	Eversion

A	SEM	3.5	5.2	3.8	2.3
	SEM × 2	7	10	8	5
B	SEM	5.4	5.3	3.3	2.3
	SEM × 2	11	11	7	5
C	SEM	4.2	5.0	2.9	2.1
	SEM × 2	8	10	6	4
